# Imaging findings in bisphosphonate-induced osteonecrosis of the jaws

**DOI:** 10.2478/v10019-010-0032-x

**Published:** 2010-06-24

**Authors:** Katarina Surlan Popovic, Miha Kocar

**Affiliations:** 1 Department of Neuroradiology, Institute of Radiology, University Medical Centre Ljubljana, Ljubljana, Slovenia; 2 Department of Maxillofacial and Oral Surgery, University Medical Centre Ljubljana, Ljubljana, Slovenia

**Keywords:** bisphosphonates, osteonecrosis, jaw, CT, MRI

## Abstract

**Background:**

Bisphosphonates are drugs used in the treatment of lytic bone metastases, multiple myeloma, hypercalcemia of malignant origin, osteoporosis, and diseases such as Paget’s disease. Recently osteonecrosis of the jaw has been associated with the use of bisphosphonates. This study describes the imaging findings of bisphosphonate-associated osteonecrosis of the jaws.

**Patients and methods:**

Eleven patients, receiving bisphosphonate medication for approximately 28 months, with pain on affected side, nonhealing extraction sockets, purulent discharge and swelling in soft tissue were examined. Imaging consisted of non-contrast enhanced CT and contrast enhanced MRI. All patients underwent surgery of affected bone and histology confirmed osteonecrosis.

**Results:**

CT scan showed osteolytic and sclerotic lesions with cortical bone destruction in all patients. The osteonecrosis was identified as delimited focal lesions with reduction of the signal on T1- weighted imaging and T2- weighted imaging. All the patients had soft-tissue involvement with enhancement in orbicular, buccinator muscle of the mouth or masticator space and adenopathy in submandibular and jugular digastric chain.

**Conclusions:**

Bisphosphonate related osteonecrosis of the jaw presents a variety of imaging findings that help to determine the extent of the disease and track the progression, however they are not specific for this disease.

## Introduction

Bisphosphonates are drugs used in the treatment of lytic bone metastases, multiple myeloma, hypercalcaemia of malignant origin, osteoporosis, and diseases such as Paget’s disease.^1^,^2^ Bisphosphonates which decrease bone turnover by inhibiting osteoclast mediated bone resorption are providing a significant improvement in the symptoms as a result of reducing pain, bone demineralization, and bone fractures, either pathologic or due to insufficiency.^2^,^3^ Given the prevalence of these diseases, bisphosphonates are one of the most prescribed drug groups in the world, particularly in patients with high incidence cancer and frequent bone metastases.^1^,^2^,^4^,^5^ Recently osteonecrosis of mandibula and maxilla has been associated with the use of biphosphonates.^6^–^10^

Such patients are referred to radiological institutes for the evaluation of bisphosphonate-induced changes in their jaws, the exclusion of other diseases of the jaws (infected osteoradionecrosis, chronic osteomyelitis or pathological fractures) and the evaluation of the jaws before orofacial procedures.^10^

The objective of this study was to examine the use of CT and MRI in the assessment of bone lesions caused by this disease.

## Patients and methods

Eleven patients, 6 men and 5 women, mean age 52 years (range 41– 76 years) who were treated with bisphosphonates were prospectively examined. They were referred to the Department of the Maxillofacial and Oral Surgery with pain on affected side, nonhealing extraction sockets, purulent discharge and swelling in soft tissue.

The indication for treatment with bisphosphonates was multiple myeloma in 6 patients (55%), breast cancer bone metastases in 3 patients (27%) and prostate cancer bone metastases in 2 patients (18%).

Nine patients were treated with zolendronic acid and two with zolendronic and pamidronic acid. The patients had taken bisphosphonate medication for approximately 28 months (range: 13– 36 months). The trigger factor in 8 patients was a tooth extraction and unknown in 3 patients. Clinical examination revealed non-healed extraction sockets in the mandibula of 6 patients and in maxilla of 2 patients. Three patients were present with pain along the mandibular nerve region and swelling of the mouth floor and in four patients palpation revealed exposed bone with irregular bone depositions. The histology of specimens showed osteonecrosis with actinomyces infection in all patients.

All patients underwent unenhanced CT and gadolinium-enhanced MRI of the jaw. CT imaging was performed in a 16-section CT machine (Somatom 16, Siemens Medical Systems, Erlangen, Germany; 45 eff. mAs, 120 kV). The CT images were reconstructed with a section thickness of 1 mm (0.6 mm increment) and multiplanar dental reconstructions were generated using commercially available dental dedicated CT software, respectively. The MRI images (3T unit; Magnetom Trio, Siemens Medical Systems) included unenhanced axial T1- weighted imaging [repetition time (TR) 470 ms, echo time (TE) 11 ms], and T2-weighted imaging (TR 6000 ms, TE 91 ms), as well as axial short-tau inversion recovery (STIR)-weighted imaging (TR 5000 ms, TE 32 ms). After an injection of contrast medium (gadolinium; Magnevist, Schering, Berlin, Germany; 0.2 mmol/kg), using a power injector, fat-saturated axial T1-weighted images (TR 612 ms, TE 11 ms) were acquired.

Two radiologists reviewed all imaging studies in consensus. In MRI studies we assessed the signal intensity change of bony structures, pathological gadolinium enhancement and soft tissue involvement. CT studies were reviewed for the presence of osteolytic and sclerotic changes of the jaw, cortical bony destruction, periostal bone proliferation and involment of inferior alveolar canal.

## Results

**FIGURE 1.**
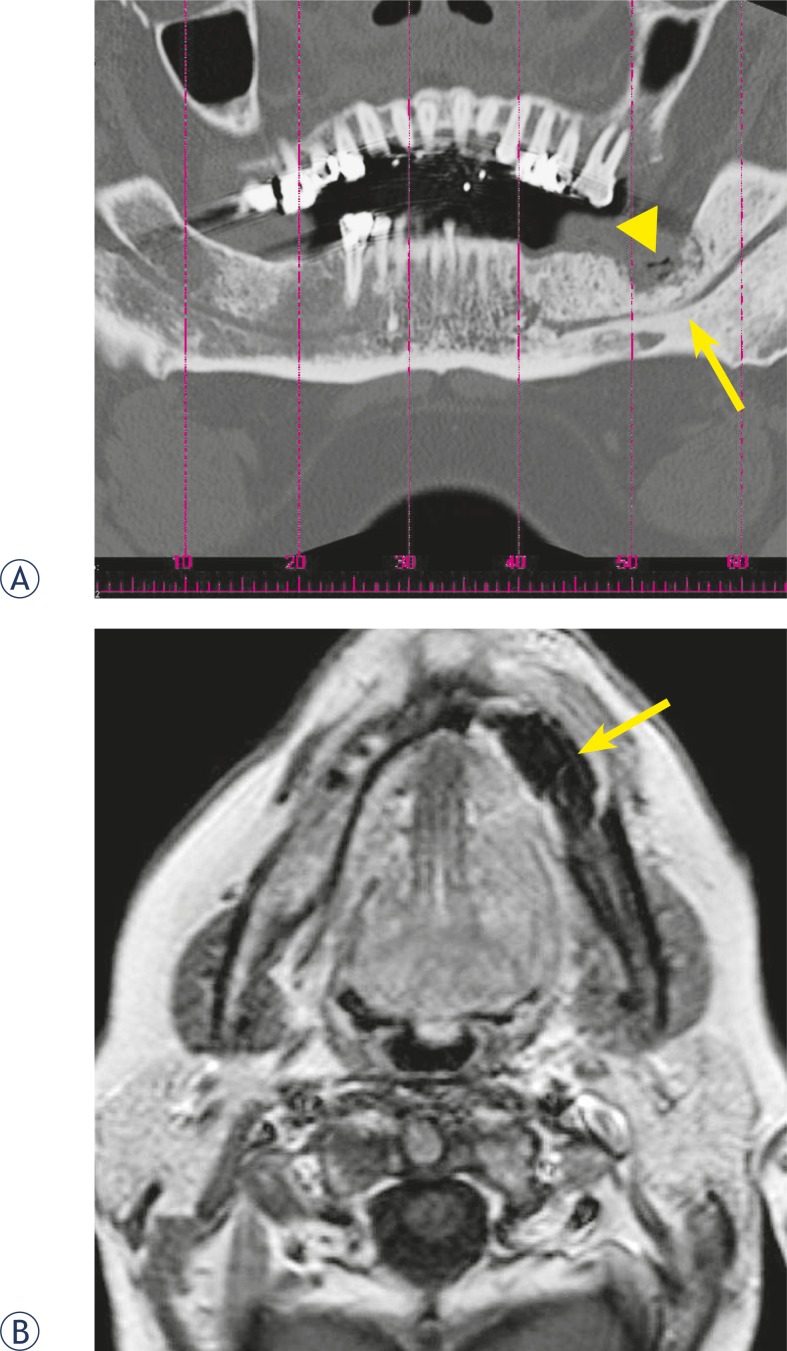
CT and MRI image of a 62-year-old woman with multiple myeloma and histologically proved bisphosphonate-induced osteonecrosis. (A) Panoramic CT reconstruction shows the osteolytic region on the left side (arrow head) as well as sclerosis of the bone marrow with involvement of the inferior alveolar canal (arrow). (B) The axial T1-weighted, contrast enhanced, MRI shows an enhancement at the periphery of the necrotic mandibular region and sequestration of mandibula (arrow).

**FIGURE 2.**
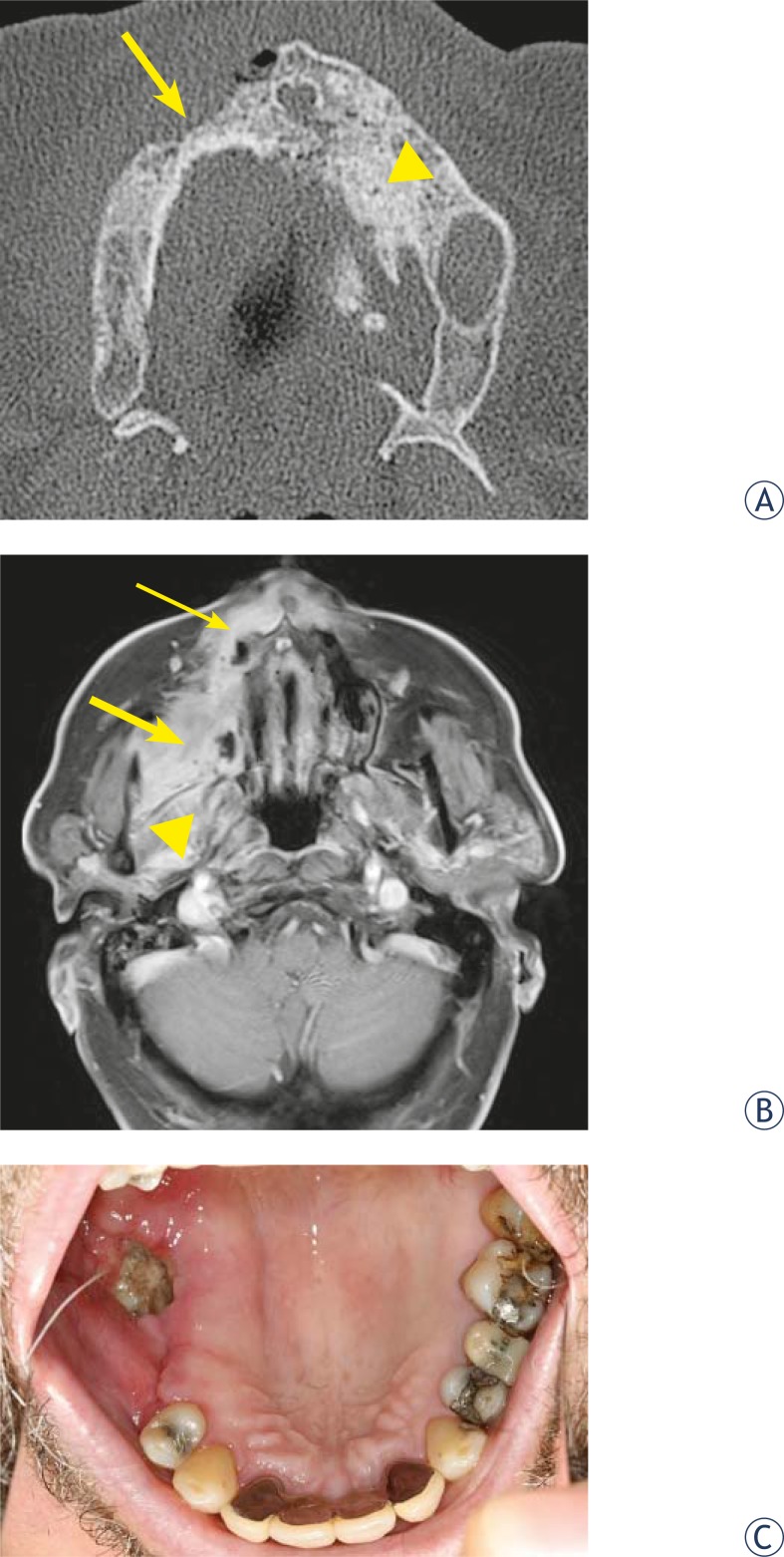
CT and MRI image of a 78-year-old man with multiple myeloma. (A) Axial CT image reveals osteolytic region in the right maxilla (arrow). There is periostal bone reaction, as well as a sclerosis of the maxilla bone marrow on the left side (arrowhead). (B) The corresponding axial T1-weighted, fat-saturated, contrast-enhanced, MRI image demonstrates enhancement in pterygoid and masseter regions (thick arrow). There is also enhancement of necrotic region in right maxilla (thin arrow) and right mandibular ramus with medial cortex resorbtion and periostal reaction (arrowhead). (C) Exposed infected (Actinomyces) bone in the same patient.

The degree of bone involvement on CT scan (Table 1) showed osteolytic and sclerotic lesions with cortical bone destruction in all patients, followed by periostal bone proliferation in 7 patients. The sclerotic change encroached on the mandibular (inferior alveolar) canal in 4 patients and 1 patient had sequestration of mandibula. On gadolinium- enhanced MRI of the jaw (Table 2) the intensity changes of the cortical and subcortical bone structures in all patients were recorded (Figures 1 and 2). The osteonecrosis was identified as delimited focal lesions with reduction of the signal on T1- weighted imaging and T2- weighted imaging. In the regions of the open wound low T1-weighted signal correlated with high T2-weighted signal. However in the regions without open wound there was no bright signal from the lesion on the T2-wighted images and enhancement after the administration of paramagnetic contrast material was significantly lower compared to the lesions with bright T2-weighted signal. All the patients had soft-tissue involvement with enhancement in orbicular, buccinator muscle of the mouth or masticator space and adenopathy in submandibular and jugular digastric chain. Maxillary sinus lesions were recorded in 2 patients.

## Discussion

Bisphosphonate-associated osteonecrosis is a new disease that is becoming increasingly more common.^1^ The adverse effect of bisphosphonat drugs was first described in 2003 by Marx^7^, Migliorati^8^ and Pogrel^9^; however, connection of phosphorus with osteonecrosis was first established in the 19^th^ century in workers of the matchmaking industry.^10^,^11^ Bisphosphonats are structurally analogous to inorganic pyrophosphates with tropism for solid calcium phosphate. Resistant to enzymatic degradation, they accumulate in bone tissue at high concentrations for long periods of time. Their mechanism of action is based on their ability to inhibit bone resorption: they increase osteoclast apoptosis while inhibiting osteocyte and osteoblast apoptosis. The addition of an amine radical increases the potency of bisphosfonat drugs. It is only with the availability of newer generation bisphosphonat drugs, the aminobisphosphonates (alendronate, ibandronate, risedronate, pamidronate, zoledronate), that side effects like osteonecrosis of the jaw have been described.^12^,^13^ The underlying pathophysiology of bosphosphonats osteonecrosis remains incompletely understood.^10^ Bisphosphonates inhibit endothelial proliferation, interrupt intraosseous circulation and bone blood flow, contributing to the development of osteonecrosis.^14^ It remains unclear whether patients receiving intravenous bisphosphonates are at a greater risk than those receiving oral bisphosphonates.^1^,^10^,^15^ Similarly, it remains unclear whether there is a predilection for the jaws. In the literature, mandibular involvement occurs in 59% of cases, maxillary involvement in 27% of cases, and combined mandibular and maxillary involvement in 8% of cases.^16^ It seems that bones exposed to constant trauma (like invasive dental procedures in the jaws) have impaired healing that may result in necrosis.^17^ Acute exacerbations of bone and soft-tissue infections are the hallmarks of osteonecrosis.^1^,^10^,^18^ While spontaneous osteonecrosis of the jaw may occur, a triggering event such as dental extraction or surgery is reported in 61.5% of cases.^13^,^19^

The American Association of Oral and Maxillofacial Surgeons has indicated that for the clinical diagnosis to be made, patients need to exhibit the following 3 criteria: *1)* current or previous treatment with a bisphosphonate; *2)* exposed, necrotic bone in the maxillofacial region that has persisted for more than 8 weeks; and *3)* no history of radiotherapy.^20^ Although the radiographic findings are not a part of the diagnostic criteria, they provide valuable information to the clinician with regard to the course, magnitude, and progression of the disease.^16^,^20^ The radiologic findings of bisphosphonate-associated osteonecrosis of the jaw are not specific and are found in other conditions such as osteomyelitis, osteoradionecrosis and cancer metastasis.^1^,^12^,^21^ CT is very useful for the ability to see and characterize the extension of the lesions and in detecting cortical involvement while MRI should be reserved for those patients who have soft tissue extension of the disease^6^, as the alteration of soft tissue is more detectable with MRI.^22^

Cross-sectional CT imaging of 11 patients from our study with initial and advanced diverse symptoms produced a range of findings, where cortical disruption with mixed lyses and sclerosis of the involved bone were the predominant imaging features. Periostal new bone formation and sequestration was recorded in the patients with advanced stage of the disease. CT finding in our study are consistent with findings in the other reports of the disease.^6^,^14^,^23^,^24^ In all our patients we detected lesions with no clinical correlation including focal sclerosis with disorganized trabeculae and difficult cortico-medullary differentiation on CT and low signal on T1 and T2 –weighted MRI images. We believe that these focal lesions are affected areas of the jaw on which a factor (*i.e.* tooth extraction) triggering the process of infection and opening up the focal lesion has not acted. This concurs with another study conducted on fourteen patients with bisphosphonate-induced osteonecrosis.^1^

In MRI the osteonecrosis appeared hypointense on T1-weighted images, but the signal intensity on T2-weighted sequence and after gadolinium enhancement varied. The lesions with intermediate signal on T2-weighted sequence showed very little contrast enhancement, which was suggestive of nonviable bone and the enhancement was probably due to inflammatory reaction caused by Actinomyces infection.^25^ Actinomyces infection was present in all specimens of our patient’s population. Same observations have been described in study of Bisdas *et al.*^10^ and Hansen *et al.*^26^ The infection was causing cervical lymphadenopathy in all patients. In patients with necrosis of mandibula pathological enhancement after contrast agent included muscles of the mouth floor, buccinator muscle and orbicular muscle, enhancement of masseter and pterygoid muscle was present in patients with maxilla and mandibular ramus necrosis. Similar MRI findings can be observed in metastatic disease, thus colleration with osseous changes on CT, clinical history and previous therapy is important for correct interpretation of MRI findings.

In conclusion, bisphosphonate related osteonecrosis of the jaw is a well described clinical condition with consistent radiographic findings; however they are not specific for this disease. It is important for radiologist to recognize this entity, because imaging could be used for early detection in patients susceptible to this disease, which means better prognosis due to early treatment, avoiding biopsy and less necessity of surgery.

## Figures and Tables

**TABLE 1.** Findings in CT imaging of the jaws of 11 patients with bisphosphonate-induced osteonecrosis of the jaws

**Findings in CT images**	**Number of patients**
Sclerotic changes	11
Osteolytic changes	11
Periostal bone proliferation	7
Sequestration	1
Inferior alveolar canal involvement	4

**TABLE 2.** Findings in contrast-enhanced imaging of the jaws of 11 patients with bisphosphonate-induced osteonecrosis of the jaws

**Findings in contrast-enhanced MRI imaging**	**Number of patients**
Intensity changes of the cortical and subcortical bone structures	11
Contrast enhancement in necrotic bone area	11
Soft-tissue involvement	11
Cervical lymphadenopathy	11
